# Regulatory Mechanisms, Protein Expression and Biological Activity of Photolyase Gene from Spodoptera littoralis Granulovirus Genome

**DOI:** 10.1007/s12033-022-00537-6

**Published:** 2022-08-18

**Authors:** Wael Elmenofy, Lamiaa El-Gaied, Reda Salem, Lamis Gomaa, Alshimaa Mahmoud, Aml Magdy, Ismail Mohamed

**Affiliations:** Agricultural Genetic Engineering Research Institute, Agricultural Research Center, Giza, 12619 Egypt

**Keywords:** *Spodoptera littoralis* granulovirus, Photolyase gene, UV irradiation, Protein expression, Transcriptional regulation

## Abstract

One of the most important factor that affects the efficient using of baculoviruses as a biopesticide is their sensitivity to UV irradiation. In this study, a photolyase gene (*phr*) of 1.4 kbp DNA fragment was cloned and characterized from *Spodoptera littoralis* granulovirus, an Egyptian isolate (SpliGV-EG1). A sequence of 466 amino acid were deduced when the gene was completely sequenced with a predicted molecular mass of ~ 55 kDa. Transcriptional regulation analyses revealed that *phr* transcripts were detected early at 6-h post-infection (hpi) and remained detectable until 72 hpi, suggesting their transcriptional regulation from a putative early promoter motif. An approximately ~ 55 kDa protein fragment was expressed from *phr*-induced bacterial culture and detected by SDS-PAGE and western blotting. In addition, direct exposure to UV irradiation resulted in a twofold decrease in SpliGV-EG1 occlusion bodies activation compared with *Spodoptera littoralis* nucleopolyhedrovirus (SpliNPV) occlusion bodies which decreased with about 129-fold after exposure to UV irradiation based on median lethal concentration value (LC50). The obtained results suggested that the presence of photolyase gene possibly alters the inactivation of SpliGV-EG1-occluded bodies by UV irradiation. These results support the role and application of the photolyase protein to improve the damaged DNA repair mechanism as well as resistance of SpliGV to UV light inactivation.

## Introduction

The baculoviruses form a family of insect-specific viruses of supercoiled dsDNA ranged from 80 to 180 kbp. The family is divided into two genera: Nucleopolyhedrovirus (NPV) and Granulovirus (GV) based on the virus shape and size [1, 2].

The NPVs subsequently divided into group I and group II based on the phylogenetic characteristics [3]. These viruses are widely used as biocontrol agents of insect pests of forest and agricultural and as vectors for the large-scale expression of foreign proteins [4]. Baculoviruses have a very narrow host range and most of them are restricted to only one insect host species. The host specificity makes them widely used for insect pest control. The viral occlusion bodies (OBs) protect the virions from environmental dissection and degradation and allow them to persist in a dormant state for a prolonged period. While OBs are stable at extreme temperatures and resistant to desiccation, the UV sensitivity forms a major limitation in large-scale application of baculoviruses in biological control. As the crystals are unable to protect baculovirus virions against ultraviolet (UV) radiation, especially from the UV-B (290–320 nm) [5]. Studies have demonstrated that baculoviruses used in the field lose the majority of their activity within 24 h of being exposed to direct sunshine [6, 7]. UV-B radiation could cause a drastic impact on the genomic viral DNA by means of forming two forms of DNA lesions: cyclobutane pyrimidine dimers (CPDs) and (6-4) photoproducts [(6-4)PPs] [6]. Lesions may interfere with polymerase activities through the DNA template, blocking both transcription and replication processes that induce mutagenesis which can cause virus attenuation due to the permanent mutation and trigger apoptosis [7]. Recombination repair, mutagenesis repair, nucleotide excision repair, and photolyase-mediated repair are some examples of various DNA damage repair methods. Among these methods, photolyase-mediated repair is the fastest and simplest DNA repair mechanism since it involves only one enzyme [6]. In addition, it is the only mechanism regulated by light [7]. Hence, the photolyases catalyze the repair of CPDs and (6-4) photoproducts by absorbing the energy between 300 and 500 nm corresponding to the near UV to blue regions.

Baculoviruses harboring a copy or more of photolyase gene are likely to live for a long time [8]. Such a gene has clear implications in insect biocontrol for their viral genomic DNA UV damage repair activity [9]. Two classes of CPD photolyases have been identified based on amino acid divergence: class I and class II CPD photolyases [9, 10]. Class II CPD photolyases are encoded by many baculoviruses [11]. The genes for baculovirus class II CPD photolyase (*phr*) were originally discovered in single nucleocapsids of Chrysodeixis chalcites nucleopolyhedrovirus (ChchNPV) and Trichoplusia ni (Tn) SNPV [12]. More recently, two class II CPD photolyase genes, assigned as phr1 and phr2, were recognized in Chrysodeixis chalcites nucleopolyhedrovirus (ChchNPV) a Dutch isolate of the baculovirus that showed 45% amino acid identity between the two Chch-PHR1 and Chch-PHR2 photolyases [9, 10]. Only single photolyase gene was identified in the genome of Trichoplusia ni (Tn) SNPV [12]. Although, complete nucleotide sequences of different NPVs and GVs genomes were reported including Chrysodeixis chalcites nucleopolyhedrovirus (ChchNPV), Trichoplusia ni NPV (T.niNPV), *Spodoptera litura* GV (SpltGV), and Spodoptera frugiperda GV (SpfrGV) [13–16], which contain a copy or more of photolyase gene. The characteristics of the photolyase gene on the molecular level and its UV damage repair activity were poorly studied and understood. The Spodoptera littoralis GV (SpliGV) is a specific potential bioagent that can be used for the specific control of *S. littoralis* population. Although, the complete nucleotide sequence of the SpliGV did not publish yet, the characteristics of the bioagent, especially its sensitivity to UV irradiation, would provide insights on the efficacy, virulence, and application of the SpliGV isolate as a promising virus-based biological control agent. In this study, we describe the characterization of Spodoptera littoralis granulovirus photolyase gene (SpliGV-phr) expression at the transcriptional and translational level as well as its biological activity upon viral occlusion bodies’ exposure to UV irradiation.

## Materials and Methods

### Insect and Viruses

The cotton leafworm, *Spodoptera littoralis* used for virus propagation, was derived from the insect-rearing facility of Agricultural Genetic Engineering Research Institute, ARC, Giza, Egypt. The larvae were maintained at 26 °C with 60% RH and reared on a semi-synthetic diet described by [17]. Virus used in this study is *S. littoralis* granulovirus (SpliGV-EG1), an Egyptian isolate.

### PCR Amplification and Sequencing of *phr*

Infected larvae were collected for virus purification and viral genomic DNA isolation using the method developed by Boughton et al. [18]. Genomic DNA was used as template for SpliGV-phr amplification. One set of phr-specific primers, designed and synthesized based on the Spodoptera litura granulovirus (SpltGV) genome sequence (accession number: NC_009503.1), was used to amplify *phr* ORF of S. littoralis granulovirus (SpliGV). Primers named Spli-phr _F (5′-GAATTCATGGATTCCACGTTCGCGCAACTACGCCAA-3′) and Spli-phr_R (5′-CTGCAG TTATTTTCTGTATTGGTTGATATAATGG-3′). The *Eco*RI and *Ps*tI restriction sites were added to the 5′ of each of the forward and reverse primer, respectively. The PCR was performed in a total reaction volume of 50 µl containing 1X Phusion® HF Buffer, 0.2 mM dNTPs, 25 pmol of each forward and reverse primers, 10 ng DNA, and 2U Phusion® DNA Polymerase. The PCR was performed under the following conditions: 98 °C for 30 s, followed by 30 amplification cycles of 98 °C for 10 s, 54 °C for 30 s, and an elongation stage of 72 °C for 1 min. Followed by final extension at 72 °C for 10 min. The produced *phr* PCR amplicon DNA was electrophoresed on a 1% (w/v) agarose gel and subsequently purified using the Qiaquick Gel purification kit (Qiagene, Germany). The purified *phr* gene fragment was cloned into pJET1.2/blunt cloning vector. The resulting vector was transformed into DH10B-Competent Cells and then extracted using a QIAGEN plasmid DNA Miniprep Kit, gel checked by electrophoresis, and sequenced using the Sanger sequence (Macrogen, Inc. Seoul, South Korean).

### Phylogenetic Analysis

The obtained *phr* gene fragment nucleotide sequence was subjected to find homologues sequences using Blastn program search data base of the National Center for Biotechnology Information (NCBI). The multiple sequence alignment and the phylogenetic tree were achieved, using Molecular Evolutionary Genetics Analysis (MEGA-X). The evolutionary history was inferred using the Neighbor-Joining method, and the evolutionary distances were computed using the p-distance method.

### Expression of PHR Protein

The amplified *phr* ORF was cloned into pQ30E expression vector using restriction enzymes *Eco*RI/*Pst*I previously added to *phr*-specific PCR primers. The cloned gene was transformed into BL21 (DE3)-competent cells and then subjected for protein induction in a time course interval using 1 mM IPTG.

### SDS-PAGE and Western Blot Analysis

Total protein purified from transformed *E. coli* was separated using 12% SDS-PAGE gel composed of 3.3 mL ddH2O, 2.5 mL of 1.5 M Tris–HCL (pH 8.8), 4 mL of acrylamide solution, 0.1 mL of 10% SDS, 0.005 m of TEMED, and 0.1 mL of 10% ammonium persulfate (APS) according to Laemmli [19]. Miniprotein II dual-slab protein gel apparatus (Bio-Rad Laboratories, USA) was used for protein separation based on protein molecular mass according to manufacturer’s instruction. The samples were loaded compared to the negative control (*E. coli* BL21 (DE3) transformed with PQE-30 empty vector). Upon completion of the run, the gels were stained using one percent of Coomassie brilliant blue solution for 2 h to show the expressed protein. For protein detection using western blotting, protein extracts were transferred onto a PVDF (polyvinylidene difluoride) membrane (MilliporeSigma Life Science Center, Germany) using a trans-blot apparatus (Bio-Rad, USA). The membrane was blocked with 5% Bovine Serum Albumin in 1X Tris-Buffered Saline and 0.1% Tween (TBS) and then washed with TBS contains 0.1%Tween-20 (TBS-T). The membrane then soaked in TBS containing anti-His-Tag antibody (1:1000) for 1 h at room temperature. Subsequently, the membrane was washed three times with TBS-T (5 min each) and kept for 1 h in TBS supplemented with anti-mouse universal antibodies (1:10,000) for 2 h. Chemofluorescence detection of His-tag protein was performed using NBT/BCIP reagent in alkaline phosphatase buffer.

### Transcriptional Analysis of *phr* Transcripts

Total RNA was isolated from the midgut of S. littoralis larvae infected with SpliGVat 6-, 12-, 24-, 48-, and 72-h post-infection using membrane-based SV Total RNA isolation purification system according to the manufacturer’s instructions (Promega, USA). Detection of *phr* transcripts using RT-PCR was carried out by Superscript III one-step RT-PCR kit (Invitrogen, Germany). The reaction was performed in 25 μl reaction volume containing 12.5 μl 2X Reaction Mix, 500 ng total RNA, 1 μl forward primer (10 μM), 1 μl reverse primer (10 μM), 1 μl RT/Platinum™ Taq Mix enzyme mix, and sterile ddH2O to 25 μl. Negative control was run with each experiment in which Superscript III RT-PCR enzyme was omitted. Thermal cycling conditions were one cycle at 60 ºC for 30 min, 94 °C for 2 min for reverse transcription, 40 cycles of three steps;15 s at 94 ºC, 30 s at 60 ºC, 40 s at 68 ºC, and final extension at 68 °C for 7 min. The cDNA mixtures were used as a template to amplify phr fragment by PCR using the gene-specific primers PI: 5′-ACGCGGGCATGAGACAGTTTC-3′ and P2: 5′-TACGGCTTCGGTGGGTTTATTTCT-3′. The obtained PCR products were analyzed in 1% (w/v) agarose gel and visualized on a UV Transilluminator.

### UV Irradiation

The occlusion bodies of SpliGV-EG1 were purified from *S. littoralis*-infected 4th instar larvae according to the method developed by Boughton et al. [18]. Briefly, SpliGV-infected S. littoralis larvae were homogenized in 0.1% SDS solution and filtered through two layers of cotton and filter paper. The filtrate was centrifuged for 15 min using 6000 xg and the pellet was re-suspended in 0.5 M NaCl and centrifuged again using the same condition. The collected pellet contains viral OBs that were finally re-suspended in suitable volume of ddH2O. One milliliter (mL) aliquots of the suspended OBs were placed in 6-well tissue culture plate and irradiated with two Philips TUVN UV-C lamps with maximum radiation at a wavelength of 254 nm for 90 s according to Petrik et al. [20]. The SpliNPV occlusion bodies, which lack a copy of photolyase gene in its genome, as a negative control was exposed to UV Irradiation and subsequently subject to LC_50_ determination using *S. littoralis* 1st instar larvae.

## Bioassays

*Spodoptera littoralis* first instar larvae were infected with a virus concentration range causing 5–95% mortality in a 7-day post-infection. For each virus concentration, minimum of 30 larvae were infected and each bioassay was three times independently repeated. The final virus concentrations used were as follows: 10^3^, 5 × 10^3^, 10^4^, 5 × 10^4^, 10^5^, and 5 × 10^5^ OBs/mL. Bioassays were performed in autoclavable 50-well plates containing 45 mL of artificial diet [21], previously mixed with 5 mL of occlusion bodies’ suspension of different concentrations per plate in independently experiments before and after exposure to UV Irradiation. Larvae that died within the first 24 h of the assay were assumed to have died from handling and were not included in the scoring. Mortality was scored on day 7 post-infection (p.i.). The EPA Probit analysis program (Version 1.5) was used for calculation of LC_50_ value before and after exposure to UV irradiation [22].

## Results

### Phylogenetic Analysis of *phr* Gene

**Fig. 1:**
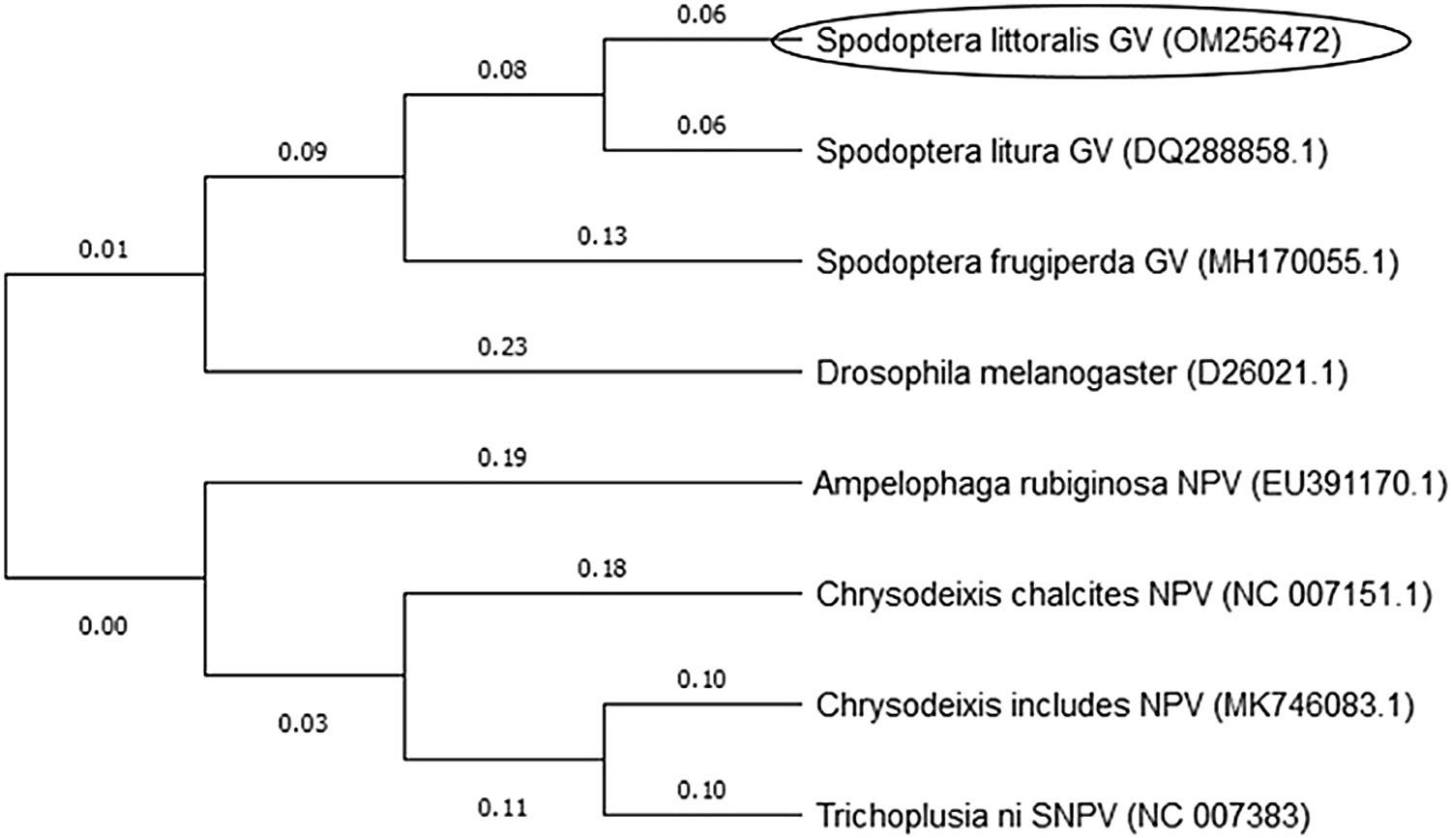
Phylogenetic analysis of *phr* nucleotide sequence of SpliGV-EG1 isolate compared to published baculoviruses *phr* sequences. Multiple sequences alignment and phylogenetic tree were generated using Clustal Omega multiple sequence alignment program (MEGA-X). Circle indicates the SpliGV-EG1 *phr* (acc. no.: OM256472)

The coding sequence of the *phr* gene was obtained from SpliGV-EG1 genomic DNA and submitted to the GenBank under accession number (OM256472), subsequently compared with other *phr* genes nucleotide sequences located in GenBank database. As shown in Fig. 1, the nucleotide sequence of SpliGV-*phr* was closed and had a common ancestor to the sequence of Spodoptera litura granulovirus (SpltGV) phr gene (Identity: 89.29%) (Accession No. DQ288858.1).

### Expression of a ~ 55 kDa Protein in pQE30 + phr-Transformed Bl21 Cells

**Fig. 2:**
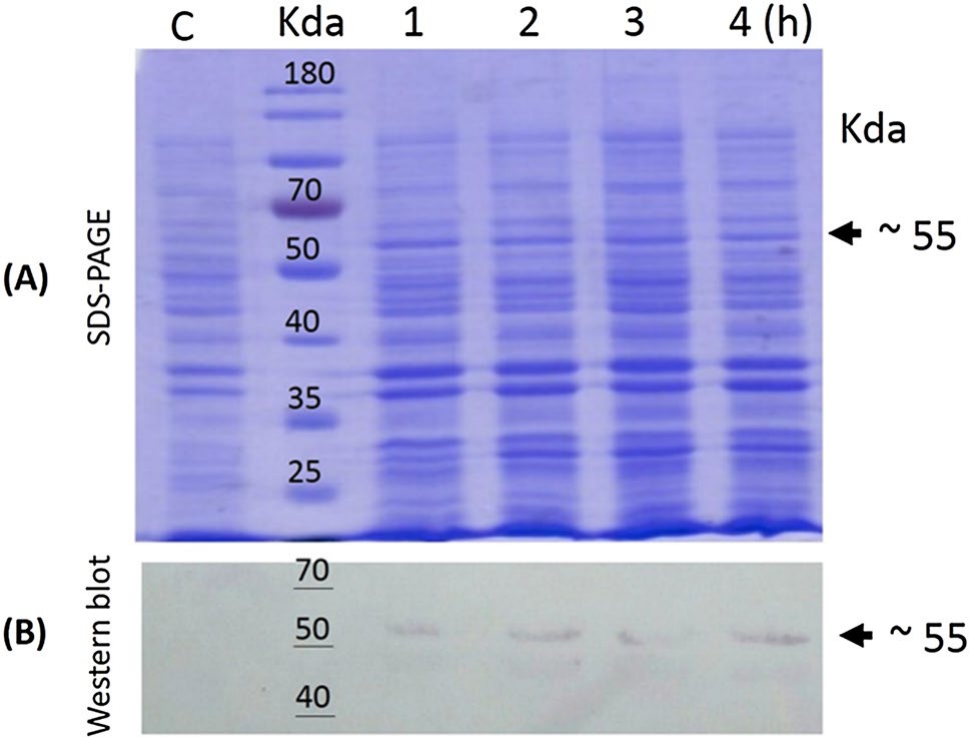
Protein expression analysis of SpliGV photolyase gene expressed in *E. coli* cells. **A** SDS-PAGE (12%) of total protein extracted from induced pQ30E vector. Lanes 1, 2, 3, and 4 (h) represent protein sampling 1-, 2-, 3-, and 4-h post-induction. C: Cells lysate extracted from empty control E.coli cells. M: Prestained protein ladder (Thermo Fisher). **B** Western blot analysis of the expressed SpliGV photolyase protein. Arrows show a clear protein band at ~ 55 kDa corresponding to Photolyase protein in both SDS-PAGE and western blot

In order to verify the phr protein molecular mass, the entire phr region from SpliGV genome of 1401 bp, presents between position 36,756 nt to position 38,156 nt, was cloned into bacterial expression vector and expressed in bacterial expression platform. The whole-cell protein analysis using SDS-PAGE revealed the appearance of ~ 55 kDa protein in pQ30E + phr construct-transformed *Escherichia coli* cells corresponding to PHR protein and absent in the empty pQ30E vector transformed *E. coli* cells. The presence of the ~ 55 kDa protein suggested the successful cloning and expression of phr region of the construct but not from the empty cloning vector. In addition, the identity of the 55-kDa polypeptide was confirmed by western blotting using His-Tag-specific monoclonal antibodies. The results of the western blotting showed that the predicted molecular mass of Photolyase protein (~ 55 kDa) was successfully detected, suggesting the correct molecular mass of Photolyase protein using the *E. coli* expression system (Fig. 2).

### RT-PCR Analysis of *phr* Transcripts

**Fig. 3:**
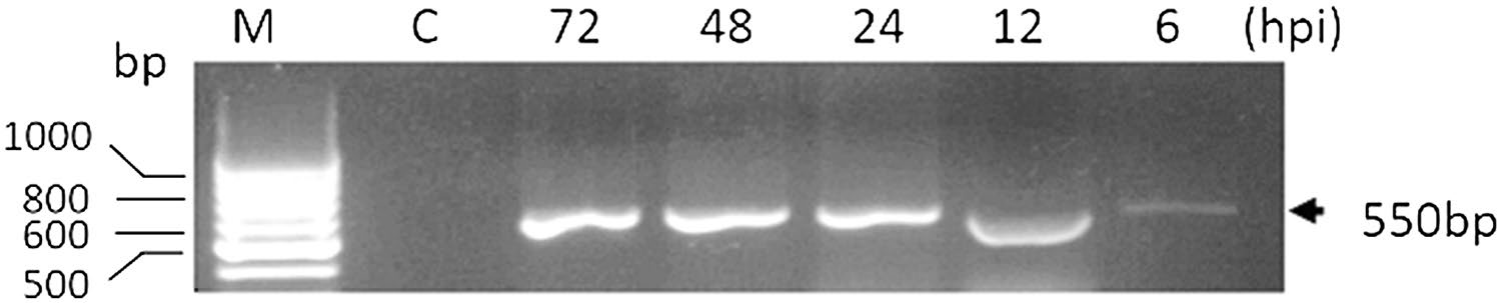
An agarose gel shows transcription regulation analysis of SpliGV-phr in S. littoralis-infected larvae using RT-PCR. Lane 1: represent −ve control in which reverse transcriptase was omitted. Lanes: 6, 12, 24, 48, and 72 represent RT-PCR product of SpliGV-infected larvae at 6-, 12-, 24-, 48-, 72-h post-infection, respectively. M: 100 bp DNA ladder

The RT-PCR analysis was applied using different time intervals to amplify a specific phr gene fragment. As shown in Fig. 3, a single band was detected with the predicted size of ~ 550 bp corresponding to phr gene (partial amplicon). The phr transcripts was first detected early at 6 hpi and remained detectable until 72 hpi.

### UV Irradiation and Bioassays

In Table 1, the obtained results showed that exposure of SpliGV Obs to UV irradiation decreased the percent mortality of *S. littoralis* 1st instar larvae after infection with SpliGV about twofold (2.9 × 10^5^ obs/mL) compared to the untreated virus Obs (1.6 × 10^5^ obs/mL) based on LC_50_ value. However, the infectivity of SpliNPV was decreased with about 129-fold (2.9 × 10^6^ obs/mL) upon exposure to UV irradiation compared to untreated SpliNPV OBs (2.3 10^4^ obs/mL). Hence, these results suggested that under these assay conditions, the presence of photolyase gene in SpliGV genome may strongly alter the inactivation of occluded virus by UV light.

**Table 1:** Analysis of the infectivity of the SpliGV & SpliNPV toward *S. littoralis* 1st instar larvae after exposure to UV Irradiation

Virus	Nr	LC50 (CL)	Slope (SE)
SpliGV before exposure to UV	559	1.6 × 10^5^ (1.1 × 10^5^ to 2.1 × 10^5^)	3.65 (0.632)
SpliGV after exposure to UV	556	2.9 × 10^5^ (1.4 × 10^5^ to 7.4 × 10^5^)	0.7 (0.15)
SpliNPV before exposure to UV	536	2.3 × 10^4^ (6.5 × 10^2^ to 6.3 × 10^4^)	1.9 (0.566)
SpliNPV after exposure to UV	606	2.9 × 10^6^ (8.9 × 10^5^ to 9.7 × 10^8^)	0.75 (0.26)

LC_50_ value was calculated as obs/mL

CL: confidence level, Nr: number of treated larvae

## Discussion

The baculovirus family (Baculoviridae) was explored for the creation of most commercial viral biopesticides among insect viruses found in nature [23, 24]. However, their UV sensitivity is a key drawback for using of the family as efficient biopesticide [25]. UV irradiation is thought to inactivate baculoviruses via sunlight by generating DNA damage as the major mechanism [26]. The photolyase enzymes known to prevent the lethal and mutagenic effects of UV irradiation [27]. In this study, the photolyase gene from SpliGV-EG1, an Egyptian isolate, was molecularly and biologically characterized. The functional characterization of *phr* of baculoviruses is critical as few reports are available from Betabaculovirus [25]. The nucleotide sequence of the complete ORF of the *phr* gene showed that it contains 1401 bp and encodes a protein of 466 amino acids with a predicted molecular mass of ~ 55 kDa. To investigate the evolutionary history of SpliGV photolyase gene, we performed a BLASTn search in the NCBI database to find similarities. We found that the SpliGV-*phr* gene is present in different baculoviruses, including members of Alphabaculovirus and Betabaculovirus. Phylogenetic evolution of the nucleotide sequence of SpliGV photolyase revealed varying levels of similarity to other baculovirus photolyases previously published in GenBank. The highest identity was 89.29% with SpltGV (accession No. DQ288858.2), and the lowest identity was 69.5% with photolyase of Chrysodeixis chalcites NPV (NC_007151.1). Interestingly, the SpliGV clade nested as a monophyletic group sharing a unique ancestor with Drosophila melanogaster (D26021.1), as well as Betabaculovirus: e.g., Spodoptera litura GV (DQ288858.1) and Spodoptera fragiperda GV (MH170055.1). Some Alpha- and Betabaculoviruses that infect members of lepidopterans showed to lack a copy of *phr* genes, including *Agrotis segetum* granulovirus [28], *Cydia pomonella* granulovirus (CpGV) [14], Xestia c-nigrum Granulovirus (XcGV) [13], as well as Spodoptera littoralis nucleopolyhedrovirus (SpliNPV) [29]. A notable trait of baculoviruses is their ability to acquire genes from insects, which includes genes involved in innate immune response [30] and apoptosis regulation [31]. Herewith the horizontal gene transfer from insects to baculovirus was reported [11]. The photolyase gene of baculoviruses showed to be acquired by alphabaculovirus and transferred to betabaculovirus [11]. On the other hand, van Oers et al. [10] described the two copies of the phr gene located in *Chrysodeixis chalcites* nucleopolyhedrovirus as a product of duplication rather than independent horizontal gene transfer. In the current study, the phr protein (PHR) was expressed using bacterial expression system and detected at ~ 55 KDa using anti-His anti-serum via western blotting, corresponding to the phr coding sequence of 1401 bp. Furthermore and in order to verify the transcriptional regulation of the photolyase mRNA, RT-PCR was performed using synthesized cDNA. The results showed that the phr transcripts could be detected from 6 to 72 h p.i., which is in agreement with it being regulated from early promoter. This is in agreement with Trichoplusia ni single-nucleopolyhedrovirus photolyase gene which was identified as an early gene product based upon promoter sequence location [12]. In addition, partial sequencing have also identified a baculovirus photolyase gene in the Chrysodeixis chalcites NPV (ChchNPV) with an early promoter motif [9].

Upon exposure of S. littoralis GV occlusion bodies to UV irradiation, the activity of the occlusion bodies was decreased against the neonates of *S. littoralis* twofold compared to the untreated SpliGV inclusion bodies. In order to verify this phenomena, these results were compared with the infectivity of SpliNPV occlusion bodies, which lack a copy of photolyase gene in its genome, before and after exposure to UV irradiation. The results showed that the infectivity was decreased with about 129-fold in comparison to untreated occlusion bodies upon exposure to the same dose of UV irradiation. This finding possibly indicates that the SpliGV harboring an active photolyase gene which increased resistance to UV damage after exposure to UV light. These results demonstrated that the SpliGV possibly able to survive longer in the field than SpliNPV. Hence, the SpliGV can be used as an enhancer of insecticidal activity of nucleopolyhedrovirus against *S. littoralis* [32]. Early research revealed that even in the absence of a true GV infection, co-feeding of NPV with GV (OBs) significantly increased the NPV's virulence and infectivity for its native host [33, 34]. Herewith co-infection between both SpliNPV and SpliGV could be a new effective strategy in order to improve the efficacy against *S. littoralis* larvae and to improve persistence of viral occlusion bodies in the environment. Thus, under these assay conditions, the results suggested that the presence of photolyase gene strongly alter the inactivation of occluded virus by UV light. It was reported that damaging effects resulted from UV irradiation may be restored via photoreactivation process upon exposure to blue light (350 –450 nm) [35]. Repairing the damage may be successfully achieved via photo reactivating enzyme photolyase. The most common type of DNA damage caused by shortwave ultraviolet (UV-C) light are the cyclobutane pyrimidine dimers (CPDs) [35]. The CPD photolyases showed to be divided into two groups (class I and II) based on the amino acid sequence similarity [36]. The S. littoralis GV photolyase is a type II CPD and homology to the reported S. litura GV [16] as well as S. frugiperda GV [37]. Despite the fact that over 50 full genomes have already been sequenced, however, photolyase genes have only been discovered in a few baculoviruses [38]. Here, a functional photolyase enzyme could be helpful to a baculovirus by enhancing viral occlusion bodies’ resistance to UV light. Upon viral infection, the infected insect larvae trying to survive longer by moving toward the top of the plant. When the cadaver’s cuticle ruptures, the viral occlusion bodies are effectively dispersed throughout the foliage, increasing the possibility of virus transmission to another larvae [39, 40]**.** Herewith the effectiveness and spreading of the virus occlusion bodies dramatically affected by exposure to UV irradiation [38]. Furthermore, transmission of viral progeny from insect to insect depends mainly on the persistence of viral occlusion bodies in the field for extended periods of time until subsequent feeding by a new insect larvae. Baculoviruses are promptly inactivated by UV radiation during this time [38]. As a result, baculoviruses are more likely to persist longer in the environment in case they have a functional photolyase gene. The UV irradiation and bioassays analysis suggested that SpliGV-*phr* gene may encodes an active photolyase, which has in vivo photolyase activity. This was presented after exposure of SpliGV to UV irradiation. As SpliGV OBs kept their efficacy against *S. littoralis* larvae even after exposure to UV light and decreased only with twofold, in comparison with SpliNPV which dramatically decreased with 129-fold upon exposure to UV irradiation using the same dose. For a UV-damaged virus to successfully commence infection, transcription, and translation of the photolyase gene must occur, the photolyase enzyme must initiate viral DNA repair, and subsequently the repair process is completed via cellular enzymes and the viral replication could continue [38]. Accordingly, improvements in the efficiency and time of repair will be required for this approach to be efficient in pest management strategies. In this case, the production of photolyase enzymes could be useful to repair thymidine dimers in viral genome. Different strategies may also be used to deliver an active photolyase to viral occlusion bodies, hence improving the viral efficacy and persistence, especially for those viruses how lack a functional repair enzyme in their genome. Hence, generation of recombinant SpliNPV that harboring SpliGV-phr may also be required in further study to examine the effect of the photolyase on the sensitivity of the SpliNPV toward direct exposure to UV irradiation.

## Conclusion

In the current study, a photolyase gene (*phr*) homolog from Spodoptera littoralis granulovirus (SpliGV-EG1) was cloned and characterized. The full-gene nucleotide sequence was determined which deduced 466 amino acids with a predicted molecular mass of ~ 55 kDa. The phylogenetic analysis confirmed the identity of the SpliGV-phr with 89.29% of similarity with Spodoptera litura granulovirus photolyase gene. The gene transcripts were detected as early as 6-h post-infection (hpi) until 72 hpi, suggesting that a putative early promoter motif was responsible for their transcriptional control. The PHR protein analysis using SDS-PAGE and western blotting showed a ~ 55 kDa protein fragment corresponding to the phr-induced protein using bacterial expression system. Upon exposure to UV irradiation, SpliGV occlusion bodies showed twofold virulence reduction toward *S. littoralis* 1st instar larvae compared to Spodoptera littoralis NPV, that lack a copy of *phr* gene, which showed 129-fold virulence reduction based on LC_50_ value. The obtained results of gene phylogeny, gene transcriptional regulation, protein expression, as well as bioassay analysis of SpliGV after exposure to UV irradiation suggested the possible enzyme activity of the SpliGV-phr and its important role in increasing the persistence of SpliGV OBs in the field under the stress of UV light inactivation.
